# Improved prognostication of glioblastoma beyond molecular subtyping by transcriptional profiling of the tumor microenvironment

**DOI:** 10.1002/1878-0261.12668

**Published:** 2020-04-04

**Authors:** Marine Jeanmougin, Annette B. Håvik, Lina Cekaite, Petter Brandal, Anita Sveen, Torstein R. Meling, Trude H. Ågesen, David Scheie, Sverre Heim, Ragnhild A. Lothe, Guro E. Lind

**Affiliations:** ^1^ Department of Molecular Oncology Institute for Cancer Research Oslo University Hospital – The Norwegian Radium Hospital Oslo Norway; ^2^ Section for Cancer Cytogenetics Institute for Cancer Genetics and Informatics Oslo University Hospital – The Norwegian Radium Hospital Oslo Norway; ^3^ Department of Oncology Oslo University Hospital – The Norwegian Radium Hospital Oslo Norway; ^4^ Institute of Clinical Medicine Faculty of Medicine University of Oslo Oslo Norway; ^5^ Department of Neurosurgery Oslo University Hospital – Rikshospitalet Oslo Norway; ^6^ Department of Pathology Copenhagen University Hospital – Rigshospitalet Copenhagen Denmark

**Keywords:** glioblastoma, infiltration, microenvironment, stratification, survival

## Abstract

Glioblastoma (GBM), the most aggressive form of brain cancer, is characterized by a high level of molecular heterogeneity, and infiltration by various immune and stromal cell populations. Important advances have been made in deciphering the microenvironment of GBMs, but its association with existing molecular subtypes and its potential prognostic role remain elusive. We have investigated the abundance of infiltrating immune and stromal cells *in silico*, from gene expression profiles. Two cohorts, including in‐house normal brain and glioma samples (*n* = 70) and a large sample set from TCGA (*n* = 393), were combined into a single exploratory dataset. A third independent cohort (*n* = 124) was used for validation. Tumors were clustered based on their microenvironment infiltration profiles, and associations with known GBM molecular subtypes and patient outcome were tested *a posteriori* in a multivariable setting. We identified a subset of GBM samples with significantly higher abundances of most immune and stromal cell populations. This subset showed increased expression of both immune suppressor and immune effector genes compared to other GBMs and was enriched for the mesenchymal molecular subtype. Survival analyses suggested that tumor microenvironment infiltration pattern was an independent prognostic factor for GBM patients. Among all, patients with the mesenchymal subtype with low immune and stromal infiltration had the poorest survival. By combining molecular subtyping with gene expression measures of tumor infiltration, the present work contributes with improving prognostic models in GBM.

## Graphical abstract

This work combined molecular subtyping of glioblastomas (GBMs) with gene expression measures of tumor infiltration and identified a subset of GBMs with high abundances of most immune and stromal cell populations. Survival analyses suggested that tumor microenvironment infiltration was an independent prognostic factor for GBM patients. Among all, patients with the mesenchymal subtype and low infiltration had the poorest survival.

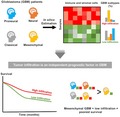

**Abbreviations**
AII: grade II astrocytoma
FDR: false discovery rate
GBM: glioblastoma
G‐CIMP: glioma CpG island methylator phenotype
GSVA: gene set variation analysis
HR: hazard ratio
IDH: isocitrate dehydrogenase
OII: grade II oligodendroglioma
pGBM: primary glioblastoma
sGBM: secondary glioblastoma
TCGA: The cancer genome atlas
WHO: World health organization

## 1 Introduction

Gliomas account for 25% of all primary central nervous system tumors in adults (Ostrom *et al.*, 2016). The World Health Organization (WHO) grading system classifies gliomas into grades I–IV based on histopathological features (Louis *et al.*, 2016). Glioblastomas (GBMs, grade IV gliomas) are the most frequent and aggressive brain tumors in adults. Lower‐grade non‐GBM tumors include grade II astrocytomas (AII) and oligodendrogliomas (OII). The least favorable prognosis is associated with GBM, which has a 5‐year survival rate around 5% (Ostrom *et al.*, 2016). While histologic classification has been a valuable tool in clinical practice, significant differences in survival among patients of the same subtype have been observed, indicating underlying clinically significant molecular heterogeneity that has not yet been detected in all its ramifications.

Advances in the understanding of glioma biology have underscored the importance of various genetic and epigenetic aberrations (Cairncross *et al.*, 1998; Gupta *et al.*, 2011; Smith *et al.*, 2000; Weller *et al.*, 2010). Among them, *IDH1/2* hotspot mutations have been observed in the vast majority of grade II/III gliomas. In high‐grade gliomas, *IDH* mutations stratify GBM into (a) primary GBMs (pGBM), *IDH* wild‐type tumors, which do not show clinical or histopathological evidence of stemming from a precursor lesion, and (b) secondary GBMs (sGBMs), which develops from lower‐grade tumors and exhibit *IDH* mutations (Gupta *et al.*, 2011; Louis *et al.*, 2016). At the transcriptome level, large efforts to establish homogenous molecular subtypes have led to the identification of four major GBM subtypes: classical, mesenchymal, proneural, and neural, which have been associated with specific genomic aberrations (Li *et al.*, 2009; Phillips *et al.*, 2006; Riddick and Fine, 2011; Verhaak *et al.*, 2010).

GBMs are also characterized by immune and stromal cell infiltration in their surrounding tumor microenvironment (Chen and Hambardzumyan, 2018; Darmanis *et al.*, 2017; Domingues *et al.*, 2016; Tomaszewski *et al.*, 2019; Wang *et al.*, 2017). Macrophages/microglia, myeloid‐derived suppressor cells, and leukocytes, mostly T‐helper cells, Tregs, and NK cells, have been consistently reported in the stroma of GBMs and associated with the main molecular subtypes (Doucette *et al.*, 2013; Gieryng *et al.*, 2017; Mirzaei *et al.*, 2017). Previous studies have focused on the prognostic value of various tumor‐infiltrating cell populations in GBMs (Becht *et al.*, 2016a; Klopfenstein *et al.*, 2019). As an example, the association of T‐cell infiltration with patient outcome has been investigated by several research groups (Han *et al.*, 2014; Kmiecik *et al.*, 2013; Mostafa *et al.*, 2016; Preusser *et al.*, 2015) and has reached inconsistent results. This underscores the complexity of the tumor microenvironment and therefore the need for further characterization. Better knowledge could improve prognostication and support qualification of GBM patients for therapies targeting the tumor microenvironment (Jackson *et al.*, 2014; Kamran *et al.*, 2017; Schaller and Sampson, 2017).

In this study, we used high‐resolution transcriptomics to analyze gene expression in glioma samples. Three independent datasets were included in the study; a series of in‐house generated expression profiles from grade II gliomas, GBMs, and normal brain samples (*n* = 70), and two publicly available GBM datasets from TCGA (*n* = 393; Brennan *et al* *.*, 2013) and GEO (*n* = 124 – http://www.ncbi.nlm.nih.gov/geo/query/acc.cgi?acc=GSE68848; Madhavan *et al.*, 2009). Particular emphasis was given to the immune and stromal characterization of the GBM microenvironment, and association with known molecular subtypes and patient outcome.

## 2 Materials and methods

### 2.1 Patients and tumor samples

The study included 67 glioma samples, which were grouped according to the 2016 revision of the WHO classification of tumors of the central nervous system (Louis *et al.*, 2016). It comprised 36 pGBMs, 5 sGBMs, 18 AIIs, and 8 OIIs from glioma patients who underwent surgery at the Department of Neurosurgery (Oslo University Hospital) between June 2006 and April 2010. Patients were included following written informed consent. Permission to include deceased patients was obtained from The National Health Authorities. The study was approved by the Regional Ethics Committee (S‐06046) as well as the Institutional Study Board. All experiments were performed in accordance with the standards set by the Declaration of Helsinki. Histological diagnoses were reviewed by an expert neuropathologist (author D.S.). Four commercially available normal brain total RNA samples were also included (BioChain, Newark, CA, USA), B209031, pool of five male donors; B306103, one male donor; Takara Bio USA (Mountain View, CA, USA), 1004311A, one male donor; Invitrogen (Waltham, MA, USA), First Choice Human Brain – 105P055201A, pool of 23 donors). The preliminary results showed that one of the normal brain samples, with material from just one donor (BioChain B306103), did not cluster with the other normal samples. As this outlier sample came from one person only, whereas the other three normal samples represented a total of 29 donors, the outlier was excluded from further analyses. The clinical characteristics of the patients and samples are summarized in Table S1.

*IDH1/IDH2* mutation analysis had previously been performed for the majority of samples (Håvik *et al.*, 2014). All *IDH* mutated samples (*n* = 29) included in our series had mutation in the *IDH1* gene, not in *IDH2*. From three samples not previously analyzed, DNA was extracted from the TRIzol left over from the RNA extraction using a standard protocol (Invitrogen). Sanger sequencing of *IDH1* and *IDH2* mutations was performed as described in the previous study from Håvik *et al. *(2014). Combined loss of 1p and 19q was examined for by loss of heterozygosity–polymerase chain reaction (LOH‐PCR) or multiplex ligation‐dependent probe amplification (MLPA) as previously described (Håvik *et al.*, 2014).

### 2.2 Microarray gene expression analysis

Tumor tissue samples were collected on RNA*later* (Qiagen, Hilden, Germany), and total RNA was extracted using a standard TRIzol protocol. Quantity and quality of RNA was assessed by NanoDrop ND‐1000 Spectrophotometer (Thermo Fisher Scientific, Waltham, MA, USA) and Agilent BioAnalyzer 2100 (Agilent Technologies, Santa Clara, CA, USA), respectively. All samples were analyzed for global gene expression using Affymetrix Human Exon 1.0. ST Array (Thermo Fisher Scientific). Total RNA (250 ng) was used as input and processed according to the manufacturer's instructions, using the Ambion WT Expression Kit protocol (Invitrogen), Affymetrix GeneChip WT terminal labeling, and Hybridization User Manual (Affymetrix, Santa Clara, CA, USA).

### 2.3 Publicly available datasets

Normalized gene expression profiles from Affymetrix H133A arrays and matching clinical information were downloaded for *n* = 441 glioblastoma samples from TCGA using the TCGA2STAT r package version 1.2 (Brennan *et al.*, 2013; Wan *et al.*, 2015). CpG island methylator phenotype (G‐CIMP) annotations were provided for each tumor. Since G‐CIMP, promoted by mutations in *IDH* (Turcan *et al.*, 2012), is associated with better outcome and younger age at diagnosis, these tumors were discarded from the dataset, as previously suggested in Klopfenstein *et al. *(2019). It resulted in *n* = 393 pGBM expression profiles. Survival data were available for all except one patient (*n* = 392), and the following treatment groups were considered for the analyses: (a) temozolomide (TMZ) chemoradiation followed by TMZ (*n* = 147), (b) radiation and TMZ (*n* = 69), or (c) radiotherapy alone (*n* = 118). Data from the Rembrandt study were included for validation purposes (Madhavan *et al.*, 2009, GEO accession number http://www.ncbi.nlm.nih.gov/geo/query/acc.cgi?acc=GSE68848). In this dataset, gene expression profiles were generated using the H133Plus2 Affymetrix platform. The 124 grade IV GBM samples were kept for the analysis, and matching clinical data were downloaded from Klopfenstein *et al. *(2019).

### 2.4 Statistical analyses and graphical representations

Statistical analyses were conducted with r version 3.2.2 and default packages from r environment. Differential analyses of expression and infiltration profiles were performed using the moderated *t*‐test approach implemented in the limma r package, version 3.30.13 (Ritchie *et al.*, 2015). All plots were created using the ggplot2 r package, version 3.0.0 (Wickham, 2016).

### 2.5 Preprocessing of the in‐house generated data

For each sample, a CEL file storing intensity measures was generated by the affymetrix genechip command console software (version 1.0). These files were further background corrected, quantile normalized, and summarized at the gene level via median polish, by the robust multi‐array average (RMA) approach using the r package oligo, version 1.34.2 (Carvalho and Irizarry, 2010). Affymetrix identifiers were converted to gene symbols from NetAffx annotation files provided by IPA (release 36).

### 2.6 Batch correction

The batch effect between the three datasets was corrected using ComBat, an empirical Bayes‐based method implemented in the SVA package (Leek *et al.*, 2012). The in‐house generated data and the TCGA expression profiles were merged to constitute the exploratory dataset (*n* = 463, among which 429 were pGBM). The third cohort was used for validation (*n* = 124). The analyses were performed on the set of 13 265 genes common to the three platforms.

### 2.7 *In silico* estimation of immune and stromal cell infiltration

Tumor infiltration by immune and other stromal cells was estimated from expression data using the MCPcounter approach, implemented in the MCPcounter r package (version 1.1.0) (Becht and de Reynies, 2016). MCPcounter provides abundance estimates for eight immune cell populations, namely CD3^+^ T cells, CD8^+^ T cells, natural killer (NK) cells, B lymphocytes, cells originating from monocytes, myeloid dendritic cells, neutrophils, and cytotoxic lymphocytes (including both CD8^+^ T cells and cytotoxic innate lymphoid NK cells). The abundance of two nonimmune stromal cell populations, that is, fibroblastic and endothelial cells, is also computed by the method. Primary GBMs were clustered based on MCPcounter abundance estimates, using a partitioning around medoids algorithm (PAM), available in the cluster r package version 2.0.3 (Maechler *et al.*, 2017). The silhouette values were computed for every sample and averaged over all data points to determine the optimal number of pGBM infiltration clusters.

### 2.8 Immune and molecular characterization of the pGBM infiltration clusters

A list of 63 key immune effector and suppressive genes, compiled by Doucette *et al. *(2013), was investigated for differential expression in the pGBM infiltration clusters and against the normal samples (see Tables S2 and S3). An FDR < 0.05 was used as the threshold for statistical significance in the various contrasts. Five representative immune gene sets, described in Thorsson *et al. *(2018), were downloaded from the paper supplementary materials. Sample‐based enrichment scores were computed using Gene Set Variation Analysis (GSVA) as implemented in the GSVA r package, for each of the five gene sets (Hänzelmann *et al.*, 2013). The clusters were also tested for association with the Verhaak's molecular subtypes (Verhaak *et al.*, 2010). The four gene sets, that is, classical, mesenchymal, neural, and proneural, were downloaded from MSigDB (Subramanian *et al.*, 2005). Primary GBM samples of the exploratory and validation cohorts were assigned a Verhaak's molecular subtype based on their highest enrichment score.

### 2.9 Survival analyses

Survival analyses were conducted with Cox's proportional hazards regression implemented in the r survival package (version 2.40‐1), with calculation of *P*‐values from Wald's tests for predictive potential (Therneau, 2015). Hazard ratios (HR) and 95% confidence intervals were derived from the model. Kaplan–Meier method was used to estimate the survival curves. Overall survival was censored at 24 months.

### 2.10 Validation

A random forest approach was implemented in order to assess the reproducibility of the clusters identified from infiltration profiles in the validation cohort, and to confirm their prognostic value. The model was trained on the MCPcounter estimates of the pGBM exploratory samples and applied to classify patients of the validation cohort. All figures included in the main paper refer to the exploratory data, and results from the validation cohort are presented in the supplementary material.

## 3 Results

### 3.1 Analyses of immune and stromal cell infiltration reveal distinct microenvironments in primary GBMs

**Fig. 1:**
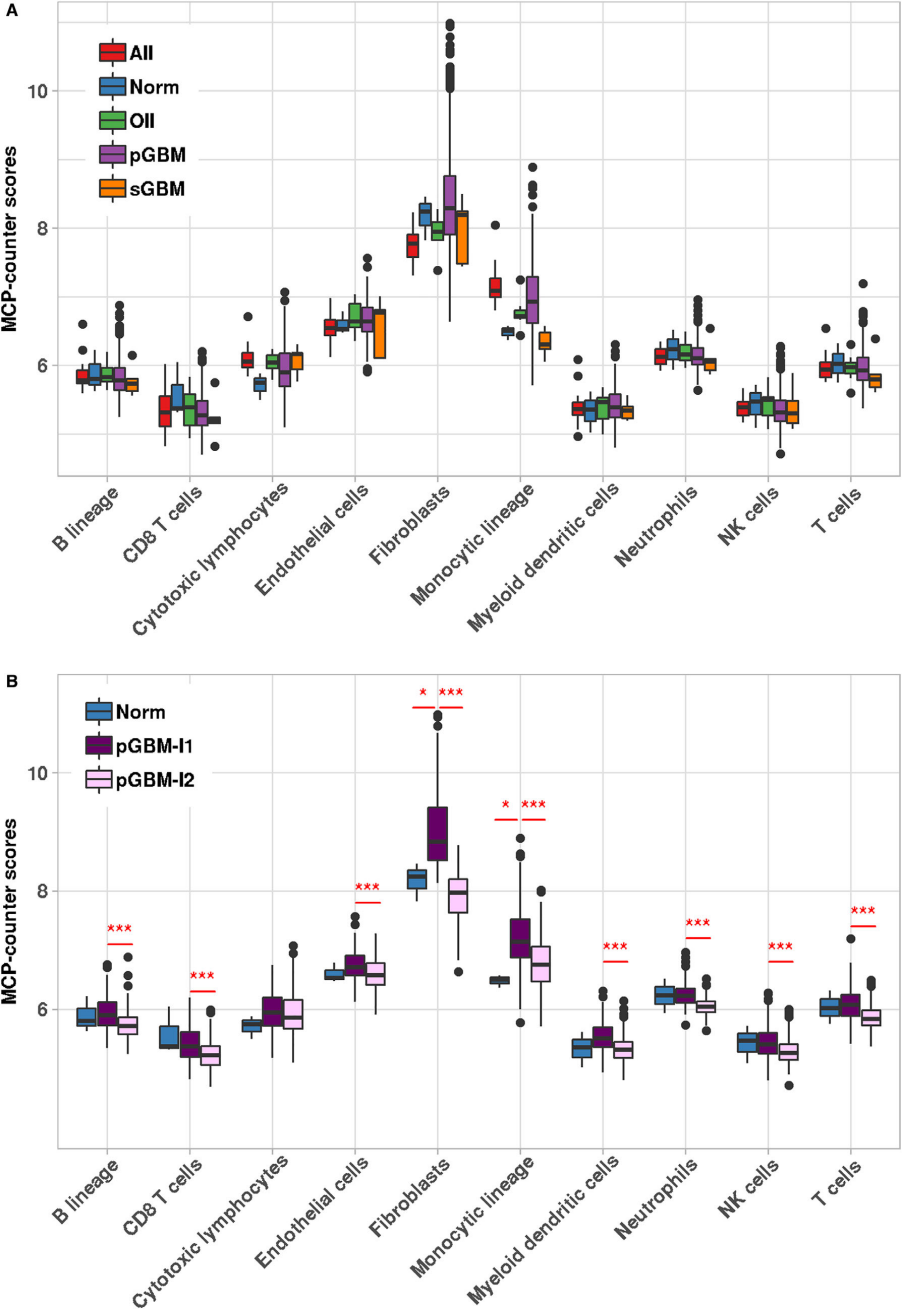
Identification of two pGBM clusters with distinct microenvironments. (A) Boxplots showing the MCPcounter abundance estimates of eight immune and two stromal cell populations among glioma subtypes (*n* = 460) and normal samples (*n* = 3) of the exploratory cohort. (B) Comparison of the MCPcounter abundance estimates in pGBM‐I1 (*n* = 192), pGBM‐I2 (*n* = 237), and normal samples (*n* = 3). For each comparison, the significance level derived from limma (FDR criterion, Benjamini–Hochberg procedure) is indicated: (*) FDR < 0.05, (**) FDR < 0.01, (***) FDR < 0.001.

*In silico* analyses of individual immune and stromal cell populations in the exploratory dataset underlined a higher variability in the composition of the microenvironment of pGBMs compared to AII and other subtypes (Fig. 1A). Using unsupervised partitioning, we conducted further investigations into the infiltration profiles of pGBMs and identified two distinct infiltration clusters of samples – denoted pGBM‐I1 (*n* = 192) and pGBM‐I2 (*n* = 237) (Fig. S1). Tumors of the pGBM‐I1 cluster showed a consistent high infiltration in fibroblasts, and moderate‐to‐high infiltration in the eight immune cell populations and endothelial cells (Fig. S2A,B). In comparison, pGBM‐I2 samples had overall lower infiltration in all immune and stromal cell populations (Fig. S2A,C). Further differential analyses established that the pGBM‐I1 cluster had significant increased infiltration in nine out of ten cell populations (B lineage, CD8 T cells, endothelial cells, fibroblasts, monocytic lineage and myeloid dendritic cells, neutrophils, NK cells, and T cells; Fig. 1B). *CD45*, a pan‐hematopoietic marker, was also considered as an alternative strategy to measure the general immune infiltration in GBMs. Its expression was highly associated with abundance of cells of monocytic origin. However, *CD45* expression poorly correlated with other immune cell populations such as NK cells or T cells (data not shown), highlighting the relevance of investigating immune cell populations individually. In the validation cohort, the random forest model trained on these nine cell populations classified the samples into pGBM‐I1 and pGBM‐I2, with significant differences in abundances between the two clusters for the same cell populations, except NK cells (Fig. S3).

**Fig. 2:**
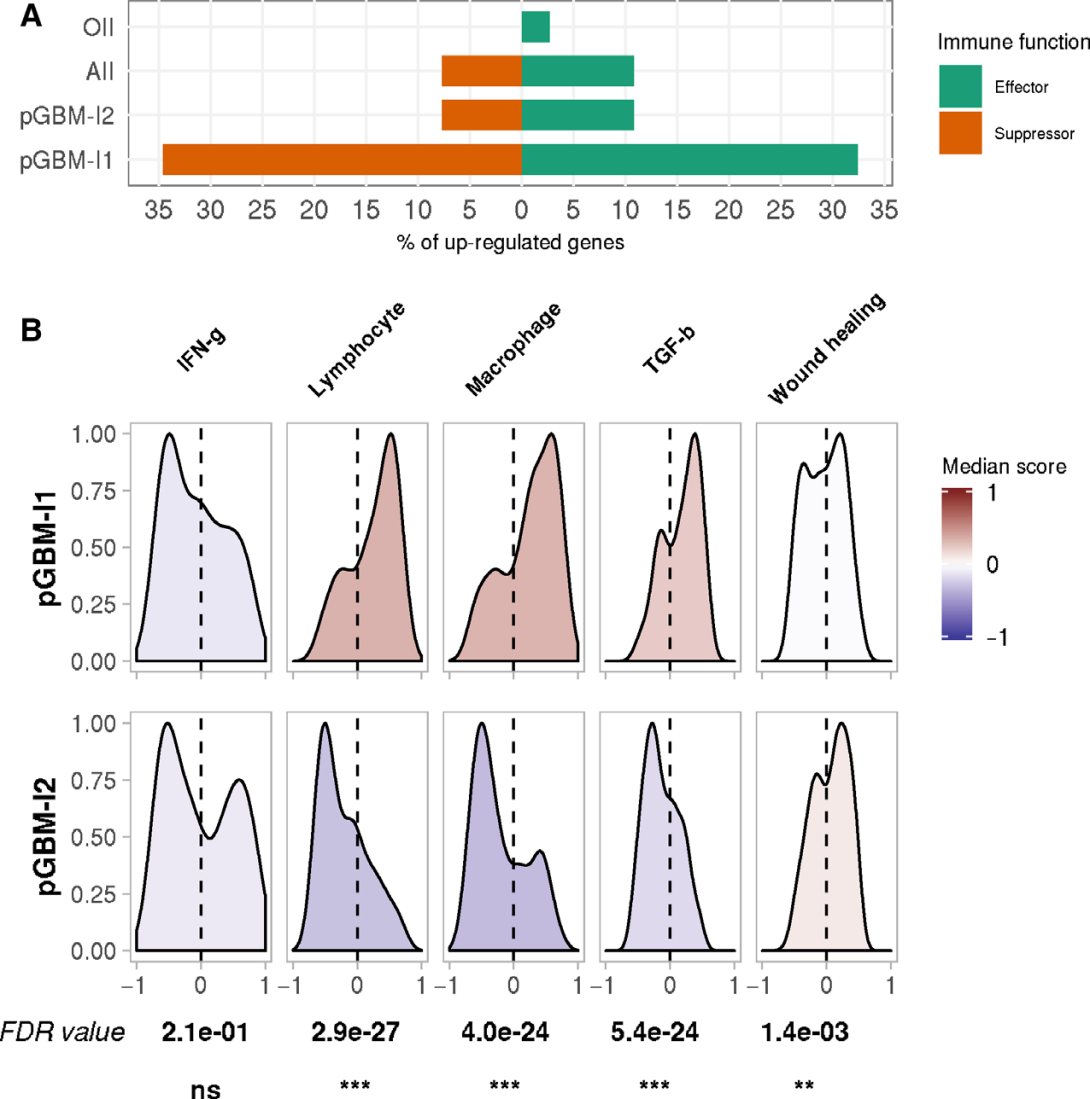
Immune characterization of the pGBM infiltration clusters. (A) Differential analyses were carried out in each glioma subtypes *vs.* normal samples in the exploratory dataset, using the moderated *t*‐test approach implemented in limma. The percentage of significantly upregulated genes (FDR < 0.05) among a list of 26 immune suppressors (red) and 37 immune effectors (green) is displayed for each glioma subtype, including the two pGBM infiltration clusters. None of the 63 genes were significantly differentially expressed in sGBM. (B) Distribution of Thorsson *et al.* signature enrichment scores among the two infiltration clusters in the exploratory dataset. The color scale indicates the median enrichment score for each gene set, with deeper red colors denoting a positive enrichment score and deeper blue colors, a negative enrichment. The gene sets were tested for differential enrichment in the two clusters. FDR values (Benjamini–Hochberg procedure) and significance levels are indicated for each comparison: (ns) not significant, (*) FDR < 0.05, (**) FDR < 0.01, (***) FDR < 0.001.

Analyzing pGBM‐I1 *vs.* pGBM‐I2 samples for differential gene expression in the exploratory dataset revealed that > 80% of the selected immune effector and suppressor genes were significantly upregulated in pGBM‐I1 (Tables S2 and S3). When tested against normal samples, pGBM‐I1 had significantly increased expression in about 35% of the suppressor genes and 32% of the effectors. The percentage of upregulated immune effectors and suppressors in pGBM‐I2 samples was comparable to AII and lower than in pGBM‐I1 (Fig. 2A). To further characterize the immune microenvironment according to the six pan‐cancer immune subtypes defined by Thorsson *et al. *(2018), we derived sample‐based enrichment scores from the five representative gene sets described in the paper: (a) activation of macrophages/monocytes, (b) overall lymphocyte infiltration, (c) TGF‐β response, (d) IFN‐γ response, and (e) wound healing (Fig. 2B). Enrichment scores were significantly higher in pGBM‐I1 for the lymphocyte, the macrophage, and the TGF‐β response signatures and to a lesser extent, significantly lower for the wound healing signature (FDR < 0.05).

### 3.2 Significant over‐representation of mesenchymal tumors in pGBM‐I1

The pGBM samples were classified according to Verhaak's molecular subtypes. The exploratory cohort included a majority of mesenchymal tumors (32%), similar rates of classical and proneural (26% and 25%) and fewer neural tumors (17%), as reported in TCGA data by Verhaak *et al. *(2010). The mesenchymal tumors displayed a significantly lower expression of *NF1* (Fig. S4), in line with the frequent number of mutations in the *NF1* tumor suppressor gene reported in the mesenchymal subtype (Verhaak *et al.*, 2010). High expressions of *CHI3L1*, *CD44*, *SERPINE1,* and *CTGF*, typical of mesenchymal tumors, were also observed (Parker *et al.*, 2016). Compared to other pGBM molecular subtypes, mesenchymal samples had increased infiltration in monocytic lineage cells (FDR = 0.0060) and a tendency toward higher infiltration in fibroblasts (FDR = 0.12) (Fig. S5).

**Fig. 3:**
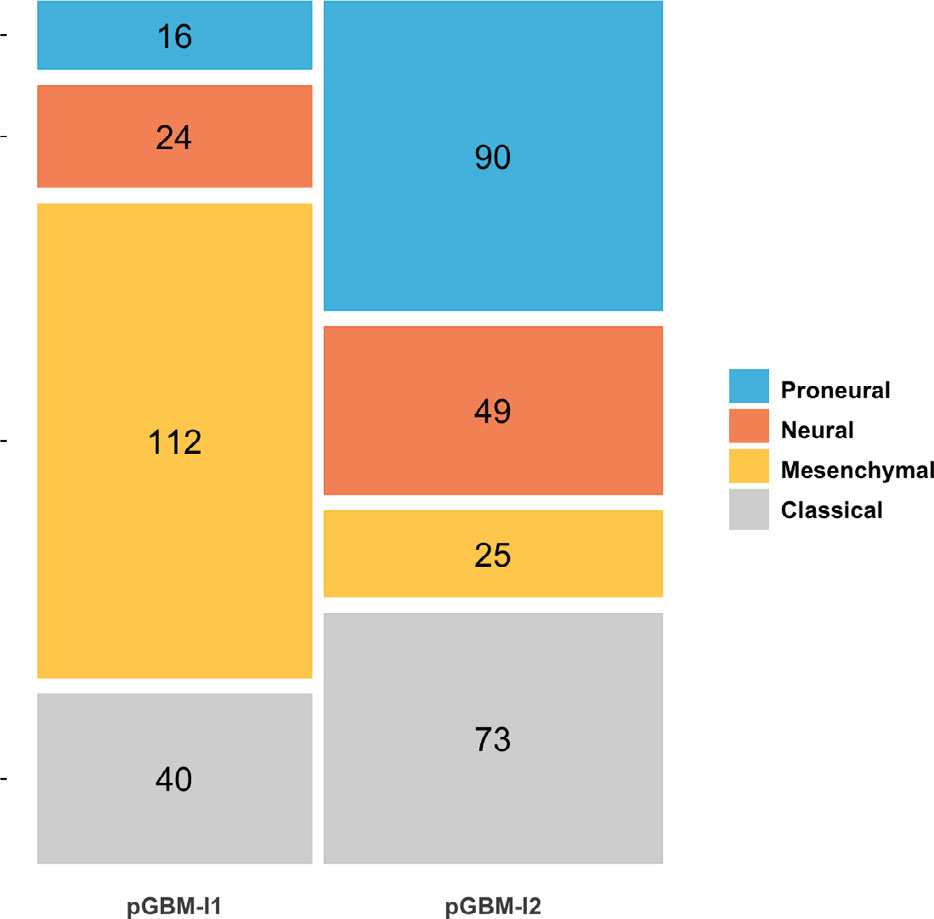
Molecular characterization of the pGBM infiltration clusters. Mosaic plot displaying the proportion of Verhaak's molecular subtypes among the pGBM‐I1 (*n* = 192) and pGBM‐I2 (*n* = 237) clusters, in the exploratory cohort. A Fisher's exact test (*P*‐value < 1e‐16) demonstrated a significant association between the molecular subtypes and the two infiltration clusters.

We found a significant association between the molecular classification and the pGBM infiltration clusters, with pGBM‐I1 being enriched in tumors of the mesenchymal subtype both in the exploratory (Fisher's exact test *P*‐value < 2e‐16, Fig. 3) and in the validation datasets (Fisher's exact test *P*‐value = 3.0e‐05, Fig. S6). The infiltration patterns observed in pGBM‐I1 and pGBM‐I2 were similar across molecular subtypes; there was a trend toward low infiltration in most cell populations in pGBM‐I2, while tumors of pGBM‐I1 had high expression in fibroblast markers and moderate‐to‐high abundance of immune cell populations and endothelial cells (Figs S7–S10).

### 3.3 Immune and stromal infiltration identifies patients with an inferior prognosis

**Fig. 4:**
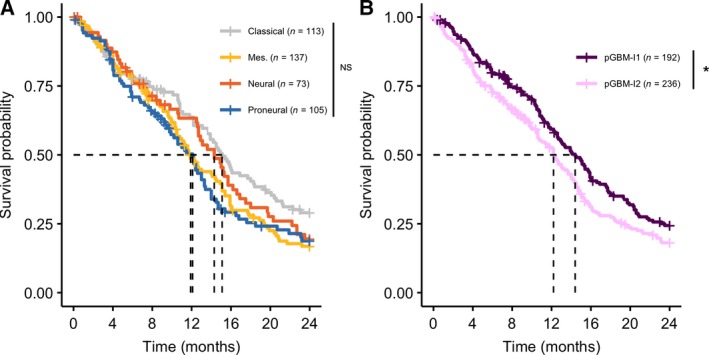
Kaplan–Meier curves modeling the effect of molecular subtypes and tumor infiltration on overall survival in pGBM patients. (A) Overall survival among pGBM patients, stratified by molecular subtypes in the exploratory cohort. The univariable Cox's regression model was overall not significant (*P*‐value = 0.086). Multivariable analyses demonstrated a significant worse prognosis for patients with the mesenchymal subtype when adjusting for the pGBM infiltration cluster [HR = 1.7 (1.2–2.4), *P*‐value = 0.0016, when compared to patients with the classical subtype]. Dashed lines are drawn at the median survival time for patient of the classical (15.1 months), mesenchymal (11.9 months), neural (14.3 months), and proneural (12.1 months) subtypes. (B) Overall survival among patients of the exploratory cohort, stratified according to the pGBM‐I1 (violet, *n* = 192) and pGBM‐I2 (pink, *n* = 236) clusters. Patients of the pGBM‐I2 subtype showed a significantly worse prognosis compared to pGBM‐I1, both in the univariable Cox's regression model [*P*‐value = 0.032 and HR = 1.3 (1.0–1.6)] and in the multivariable model including the tumor molecular subtype and infiltration cluster annotation as covariates [*P*‐value = 0.0037 and HR = 1.5 (1.1–2.0)]. Dashed lines are drawn at the median survival time for pGBM‐I1 (14.4 months) and pGBM‐I2 patients (12.2 months). *P*‐values are derived from Cox's regression models and hazard ratio (HR) are provided together with their 95% confidence interval. The significance levels of the univariable models are displayed on the figures. Abbreviations: (NS) not significant, (*) *P*‐value < 0.05.

We tested the overall significance of several univariable Cox's regression models to predict patient survival in the exploratory cohort. The regression model including the Verhaak's molecular classification alone as a factor did not reach the significance level (Wald's test *P*‐value = 0.086, Fig. 4A, Table S4). In comparison, the univariable model accounting for the infiltration clusters was overall significant, with pGBM‐I2 being associated with worse prognosis compared to pGBM‐I1 [Wald's test *P*‐value = 0.032, HR = 1.3 (1.0–1.6), Fig. 4B]. We found no association between the pGBM infiltration clusters and the type of treatment received after surgery (Fisher's exact test *P*‐value = 0.36). A multivariable Cox's regression model, including the molecular subtypes and the infiltration cluster annotation as covariates, was fitted to the data. Comparing the uni‐ and multivariable models using deviance and chi‐square statistic revealed that adding the infiltration clusters significantly improved our ability to predict survival compared to the molecular subtypes alone (*P*‐value = 0.0034). We also tested whether we should account for potential differences in survival in our own series and the TCGA series, but no significant effect was observed. The molecular subtypes and infiltration clusters had independent prognostic values in the multivariable model (Table S4). When controlling for the molecular subtype, pGBM‐I2 was significantly associated with inferior survival compared to pGBM‐I1 [HR = 1.5 (1.1–2.0), *P*‐value = 0.0037]. Mesenchymal tumors showed significantly worse outcome among pGBM patients when adjusting for immune and stromal infiltration [HR = 1.7 (1.2–2.4), *P*‐value = 0.0016]. Patient with mesenchymal pGBM‐I2 tumors had the worst prognosis, with a median survival < 10 months and an HR = 3.0 (1.7–5.4) (*P*‐value = 2.5e‐4) when compared to classical pGBM‐I1, which had the best outcome (median survival = 18 months).

In the validation cohort, the proneural subtype was significantly associated with a better outcome [*P*‐value = 0.018, HR = 0.42 (0.21–0.86), Fig. S11 and Table S5]. The survival between pGBM‐I1 and pGBM‐I2 patients was not significantly different in the univariable model [*P*‐value = 0.47 and HR = 1.2 (0.75–1.9), Fig. S12 and Table S5]. Including the immune and stromal infiltration as a covariate, in addition to the molecular subtype, significantly improved the fit of the Cox's regression. We confirmed the independent prognostic value of the molecular subtype and infiltration cluster in the multivariable model; patients with pGBM‐I2 tumors were found to have a significantly worse outcome compared to pGBM‐I1 [*P*‐value = 0.048, HR = 1.7 (1.0–2.8), Table S5]. Mesenchymal pGBM‐I2 had again the shortest median survival (11 months).

Combining survival data from the exploratory and validation cohorts strengthened our finding and confirmed the independent prognostic value of the infiltration clusters (Table S6). In stratified analyses, the mesenchymal subtype showed consistently significantly worse outcome among patients with high (pGBM‐I1) or low (pGBM‐I2) tumor infiltration (Table S7 and Fig. S13).

## 4 Discussion

Gene expression profiling was used to investigate the abundance of tumor‐infiltrating immune and stromal cells in gliomas. Our study revealed a substantial variability in the composition of the microenvironment of pGBMs compared to low‐grade gliomas, in line with the findings of Doucette *et al.* (2013) and Wang *et al.* (2017). Two clusters of pGBM samples, called pGBM‐I1 and pGBM‐I2 and displaying distinct tumor microenvironments, were identified from the exploratory cohort and successfully validated in an independent dataset. The clusters were characterized based on (a) single pro‐inflammatory and immune suppressive genes selected from Doucette *et al. *(2013), (b) previously established immune signatures (Thorsson *et al.*, 2018), and (c) abundance of individual tumor‐infiltrating immune and stromal cell populations (Becht and de Reynies, 2016). Finally, we assessed the performance of a survival model combining molecular and tumor microenvironment classifications, in predicting prognosis of GBM patients.

Tumors of pGBM‐I1 displayed higher expression in most of the pro‐inflammatory and immune‐suppressive genes compared to pGBM‐I2. When analyzed for activation of the representative gene sets previously reported in Thorsson *et al.* (2018), the clusters showed significant differential enrichment for most of the sets. Based on expression data, tumors of pGBM‐I2 had a profile similar to the ‘lymphocyte‐depleted’ subtype established by Thorsson *et al. *(2018) and described as being enriched in glioma samples. With an overall higher activation of the TGF‐β, macrophage, and lymphocyte signatures, the pGBM‐I1 cluster was, on the other hand, closer to the ‘TGF‐β‐dominant’ immune subtype. Interestingly, the ‘lymphocyte‐depleted’ and ‘TGF‐β‐dominant’ immune subtypes were both reported by Thorsson *et al. *(2018) to have the least favorable outcome among all the pan‐cancer subtypes, a finding in accordance with the overall poor prognosis of GBMs. Tumors of pGBM‐I1 were characterized by high expression of fibroblast markers, and moderate‐to‐high infiltration in immune cell populations and endothelial cells, and were associated with the mesenchymal subtype. They also displayed significantly higher expression of immune checkpoints such as *PDL1*, in line with Liu *et al. *(2017), and *CTLA4,* indicating they could be good candidates for immune checkpoint inhibitors therapies. Although others have previously reported similar finding in mesenchymal tumors (Chen and Hambardzumyan, 2018; Doucette *et al.*, 2013; Kaffes *et al.*, 2019), we provide here an alternative strategy based on unsupervised learning, without making any prior assumption on the tumor molecular subtypes. Our approach enables to identify tumors from other molecular subtypes presenting a similar immune and stromal phenotype, which could also benefit from treatments targeting the microenvironment. Interestingly, the distinct makeup found in the microenvironment of mesenchymal pGBM has also been described in mesenchymal colorectal tumors (Becht *et al.*, 2016b), suggesting that similar patient stratification and therapeutic strategies could be implemented across cancer types.

When it comes to survival, studies have reported different clinical outcomes for pGBM patients. It has been proposed that proneural tumors are associated with increased survival compared to other molecular subtypes, while mesenchymal tumors have an inferior outcome (Phillips *et al.*, 2006). In our exploratory cohort, a trend for worse prognosis in both the mesenchymal and the proneural subtypes was observed, but overall the molecular classification was not significantly associated with patient's survival in a univariable setting. In the validation cohort, the proneural subtype was significantly associated with better outcome as also demonstrated in Klopfenstein *et al. *(2019). Others have investigated the prognostic value of various tumor‐infiltrating cell populations in GBM. Using univariable analyses, Becht *et al. *(2016a) reported that infiltration in most immune cell populations was associated with improved survival, while increased fibroblast infiltration had a negative prognostic value. In another study, Busek *et al. *(2016) documented no association between expression of a marker of cancer‐associated fibroblast and survival. They speculated that the glioma subtype may act as a confounder and could explain the inconsistent results regarding the prognostic value of fibroblasts in the literature. These discrepancies suggest that when considered alone, molecular subtypes or immune infiltration does not fully capture the varied clinical course of pGBMs. Our work established that both factors have independent prognostic value and combining them significantly improved our ability to predict survival, as also reported by others (Gruosso *et al.*, 2019; Zeng *et al.*, 2019), including recently in GBM (Klopfenstein *et al.*, 2019).

## 5 Conclusions

In the present study, transcriptomic profiling identified GBM samples with distinct infiltration of immune and stromal cell populations. Our findings could be further exploited to support qualification of patients for therapeutic strategies targeting the tumor microenvironment. We also established that estimations of immune and stromal infiltration combined with known molecular subtypes improve prognostic models in GBM.

## Conflict of interest

The authors declare no conflict of interest.

## Author contributions

GEL, RAL, PB, and SH were responsible for the conception and design. ABH, PB, TRM, THA, and DS were responsible for the acquisition of data. MJ, ABH, LC, AS, and GEL were responsible for the analyses and interpretation of data. MJ has drafted the manuscript. All authors read and approved the final manuscript.

## Supporting information


**Fig. S1.** Selection of the number of pGBM infiltration clusters in the exploratory cohort.
**Fig. S2.** Estimation of tumor infiltration across samples of the exploratory cohort.
**Fig. S3.** Differential abundance of immune and stromal cell populations between pGBM‐I1 and pGBM‐I2, in the validation dataset.
**Fig. S4.** Expression levels of selected genes in the mesenchymal subtype *vs.* other subtypes (neural, proneural and classical), in the exploratory cohort.
**Fig. S5.** Comparison of abundance of immune and stromal cell populations in the pGBM molecular subtypes (exploratory cohort).
**Fig. S6.** Molecular characterization of the pGBM‐I1 and pGBM‐I2 clusters according to the Verhaak's signatures in the validation dataset.
**Fig. S7.** Heatmaps of MCPcounter scores in mesenchymal tumors of the exploratory cohort.
**Fig. S8.** Heatmaps of MCPcounter scores in neural tumors of the exploratory cohort.
**Fig. S9.** Heatmaps of MCPcounter scores in classical tumors of the exploratory cohort.
**Fig. S10.** Heatmaps of MCPcounter scores in proneural tumors of the exploratory cohort.
**Fig. S11.** Kaplan‐Meier curves modeling the effect of GBM molecular classification on survival in the validation cohort.
**Fig. S12.** Kaplan‐Meier curves modeling the effect of tumor infiltration on overall survival in the validation cohort.
**Fig. S13.** Effect of molecular subtypes on survival, in the pooled exploratory and validation cohorts, in (A) pGBM‐I1 and (B) pGBM‐I2.
**Table S1.** Summary of in‐house patient characteristics (n=67).
**Table S2.** Regulation of immune suppressor genes in the pGBM infiltration clusters of the exploratory cohort.
**Table S3.** Regulation of immune effector genes in the pGBM infiltration clusters of the exploratory cohort.
**Table S4.** Univariable and multivariable Cox's proportional hazard analyses, with survival as endpoint, in the exploratory cohort.
**Table S5.** Univariable and multivariable Cox's proportional hazard analyses, with survival as endpoint, in the validation cohort.
**Table S6.** Univariable and multivariable Cox's proportional hazard analyses, with survival as endpoint, in the pooled exploratory and validation cohorts.
**Table S7.** Effect of molecular subtypes on survival in pGBM‐I1 and pGBM‐I2, in the pooled exploratory and validation cohorts.

## Data Availability

The in‐house generated dataset is available in the GEO repository (GEO: http://www.ncbi.nlm.nih.gov/geo/query/acc.cgi?acc=GSE111260).
