# Supplementary material for: Improved prognostication of glioblastoma beyond molecular subtyping by transcriptional profiling of the tumor microenvironment
Source: Mol Oncol. 2020 Apr 4;14(5):1016–27. doi: 10.1002/1878-0261.12668 (PMC7191188; doi:10.1002/1878-0261.12668)
Supplement: Supplementary file 1 — Fig. S1. Selection of the number of pGBM infiltration clusters in the exploratory cohort. Fig. S2. Estimation of tumor infiltration across samples of the exploratory cohort. Fig. S3. Differential abundance of immune and stromal cell populations between pGBM‐I1 and pGBM‐I2, in the validation dataset. Fig. S4. Expression levels of selected genes in the mesenchymal subtype vs. other subtypes (neural, proneural and classical), in the exploratory cohort. Fig. S5. Comparison of abundance of immune and stromal cell populations in the pGBM molecular subtypes (exploratory cohort). Fig. S6. Molecular characterization of the pGBM‐I1 and pGBM‐I2 clusters according to the Verhaak's signatures in the validation dataset. Fig. S7. Heatmaps of MCPcounter scores in mesenchymal tumors of the exploratory cohort. Fig. S8. Heatmaps of MCPcounter scores in neural tumors of the exploratory cohort. Fig. S9. Heatmaps of MCPcounter scores in classical tumors of the exploratory cohort. Fig. S10. Heatmaps of MCPcounter scores in proneural tumors of the exploratory cohort. Fig. S11. Kaplan‐Meier curves modeling the effect of GBM molecular classification on survival in the validation cohort. Fig. S12. Kaplan‐Meier curves modeling the effect of tumor infiltration on overall survival in the validation cohort. Fig. S13. Effect of molecular subtypes on survival, in the pooled exploratory and validation cohorts, in (A) pGBM‐I1 and (B) pGBM‐I2. Table S1. Summary of in‐house patient characteristics (n=67). Table S2. Regulation of immune suppressor genes in the pGBM infiltration clusters of the exploratory cohort. Table S3. Regulation of immune effector genes in the pGBM infiltration clusters of the exploratory cohort. Table S4. Univariable and multivariable Cox's proportional hazard analyses, with survival as endpoint, in the exploratory cohort. Table S5. Univariable and multivariable Cox's proportional hazard analyses, with survival as endpoint, in the validation cohort. Table S6. Univariable and multi [file MOL2-14-1016-s001.pdf]

Supplementary material

Content

i. Tables

- **Supplementary Table 1 (pages 5-9)**

Summary of in-house patient characteristics (n=67)

- **Supplementary Table 2 (pages 10-11)**

Regulation of immune suppressor genes in the pGBM infiltration clusters of the exploratory cohort.

- **Supplementary Table 3 (pages 12-14)**

Regulation of immune effector genes in the pGBM infiltration clusters of the exploratory cohort.

- **Supplementary Table 4 (page 15)**

Univariable and multivariable Cox's proportional hazard analyses, with survival as endpoint, in the exploratory cohort.

- **Supplementary Table 5 (page 16)**

Univariable and multivariable Cox's proportional hazard analyses, with survival as endpoint, in the validation cohort.

- **Supplementary Table 6 (page 17)**

Univariable and multivariable Cox's proportional hazard analyses, with survival as endpoint, in the pooled exploratory and validation cohorts.

- **Supplementary Table 7 (page 18)**

Effect of molecular subtypes on survival in pGBM-I1 and pGBM-I2, in the pooled exploratory and validation cohorts

ii. <u>Figures</u>

- **Supplementary Figure 1 (page 19)**

Selection of the number of pGBM infiltration clusters in the exploratory cohort.

- **Supplementary Figure 2 (page 20)**

Estimation of tumor infiltration across samples of the exploratory cohort.

- **Supplementary Figure 3 (page 21)**

Differential abundance of immune and stromal cell populations between pGBM-I1 and pGBM-I2, in the validation dataset.

- **Supplementary Figure 4 (page 22)**

Expression levels of selected genes in the mesenchymal subtype *vs.* other subtypes (neural, proneural and classical), in the exploratory cohort.

- **Supplementary Figure 5 (page 23)**

Comparison of abundance of immune and stromal cell populations in the pGBM molecular subtypes (exploratory cohort).

- **Supplementary Figure 6 (page 24)**

Molecular characterization of the pGBM-I1 and pGBM-I2 clusters according to the Verhaak's signatures in the validation dataset.

- **Supplementary Figure 7 (page 25)**

Heatmaps of MCPcounter scores in mesenchymal tumors of the exploratory cohort.

- **Supplementary Figure 8 (page 26)**

Heatmaps of MCPcounter scores in neural tumors of the exploratory cohort.

- **Supplementary Figure 9 (page 27)**

Heatmaps of MCPcounter scores in classical tumors of the exploratory cohort.

- **Supplementary Figure 10 (page 28)**

Heatmaps of MCPcounter scores in proneural tumors of the exploratory cohort.

- **Supplementary Figure 11 (page 29)**

Kaplan-Meier curves modeling the effect of GBM molecular classification on survival in the validation cohort.

- **Supplementary Figure 12 (page 30)**

Kaplan-Meier curves modeling the effect of tumor infiltration on overall survival in the validation cohort.

- **Supplementary Figure 13 (pages 31)**

Effect of molecular subtypes on survival, in the pooled exploratory and validation cohorts, in (A) pGBM-I1 and (B) pGBM-I2.

Sample ID	Gender	Age[c]	Histopathological diagnosis	WHO grade	*IDH1/IDH2* status	Combined +7-10[d]	LOH 1p/19q	*MGMT* promoter methylation[e]
06-1563[a]	Male	35	A	II	MUT	NA	No	U/M
07-0658	Male	25	A	II	MUT	NA	No	U/M
07-0841	Male	32	A	II	MUT	NA	No	NA
07-1082	Male	28	A	II	MUT	NA	No	U/M
08-0706	Female	42	A	II	MUT	NA	No	U/M
08-1168	Female	43	A	II	MUT	NA	No	U/M
08-1169	Male	37	A	II	MUT	NA	No	M/M
1002-0669	Male	31	A	II	MUT	NA	No	NA
07-0025	Male	64	A	II	MUT	NA	No	U/M
07-0041[b]	Female	29	A	II	MUT	NA	No	U/M
07-0139	Male	40	A	II	MUT	NA	No	M/M
07-0350	Male	35	A	II	MUT	NA	No	M/M

07-0372	Female	32	A	II	MUT	NA	No	M/M
07-0450	Male	36	O	II	MUT	NA	Yes	M/M
07-0697	Male	51	A	II	MUT	NA	No	M/M
08-1321	Female	41	A	II	MUT	NA	No	NA
08-1498	Female	61	A	II	MUT	NA	No	U/M
08-1510	Male	38	A	II	MUT	NA	No	U/M
08-1592	Male	55	O	II	MUT	NA	Yes	M/M
06-1206	Female	36	O	II	MUT	NA	Yes	M/M
06-1467	Female	30	O	II	MUT	NA	Yes	U/M
07-0704	Male	45	O	II	MUT	NA	Yes	U/M
07-1341	Male	23	O	II	MUT	NA	Yes	U/M
07-1656	Female	51	A	II	WT	NA	No	U/U
09-0349	Female	36	O	II	NA	NA	Yes	NA
1002-0413	Female	40	O	II	MUT	NA	Yes	NA
06-0811	Male	70	pGBM	IV	WT	NA	NA	U/U
06-0813	Female	82	pGBM	IV	NA	NA	NA	NA
06-0823	Male	76	pGBM	IV	WT	Yes	NA	M/M

06-0849	Male	56	pGBM	IV	WT	Yes	NA	U/U
06-0984	Male	63	pGBM	IV	WT	No	NA	M/M
06-1051	Male	61	pGBM	IV	WT	Yes	NA	U/U
06-1126	Female	59	pGBM	IV	WT	Yes	NA	M/M
06-1163	Female	84	pGBM	IV	NA	NA	NA	NA
06-1277	Male	70	pGBM	IV	WT	Yes	NA	U/U
06-1471	Female	79	pGBM	IV	WT	No	NA	M/M
06-1591	Female	58	pGBM	IV	WT	Yes	NA	U/M
06-1603	Male	53	pGBM	IV	WT	Yes	NA	U/M
07-0173	Female	46	pGBM	IV	WT	Yes	NA	U/U
07-0666	Male	58	pGBM	IV	NA	No	NA	NA
07-0860	Male	65	pGBM	IV	WT	Yes	NA	U/U
07-0968	Male	62	pGBM	IV	WT	Yes	NA	U/M
07-1024	Male	81	pGBM	IV	WT	NA	NA	NA
07-1380	Male	70	pGBM	IV	WT	Yes	NA	M/M
07-1562	Male	56	pGBM	IV	WT	NA	NA	U/U
07-1568	Male	55	pGBM	IV	WT	No	NA	M/M

08-0123	Female	54	pGBM	IV	WT	NA	NA	M/M
08-0193	Male	48	pGBM	IV	WT	NA	NA	U/U
08-0194	Female	65	pGBM	IV	WT	NA	NA	M/M
08-0269	Male	59	pGBM	IV	WT	NA	NA	U/U
08-0308	Female	36	pGBM	IV	WT	NA	NA	U/U
08-0315	Female	61	pGBM	IV	WT	NA	NA	M/M
08-0316	Female	81	pGBM	IV	WT	NA	NA	NA
08-0417	Female	63	pGBM	IV	WT	NA	NA	M/M
08-0723	Female	56	pGBM	IV	WT	NA	NA	U/U
08-1253	Male	50	pGBM	IV	WT	NA	NA	U/U
08-1516	Male	81	pGBM	IV	WT	NA	NA	U/U
08-1556	Male	75	pGBM	IV	WT	NA	NA	U/U
09-0133	Male	55	pGBM	IV	WT	NA	NA	M/M
09-0201	Female	67	pGBM	IV	WT	NA	NA	NA
0992-0111	Female	82	pGBM	IV	WT	NA	NA	NA
0992-0314	Female	56	pGBM	IV	WT	NA	NA	NA
06-1223	Male	46	sGBM	IV	MUT	Yes	NA	M/M

07-0196	Male	48	sGBM	IV	MUT	NA	NA	U/M
08-0243	Female	38	sGBM	IV	MUT	NA	NA	NA
09-0194 [a]	Male	38	sGBM	IV	MUT	NA	NA	NA
09-0380 [b]	Female	32	sGBM	IV	MUT	NA	NA	NA

[a] Samples originate from the same patient. [b] Samples originate from the same patient. [c] Age in years, [d] combined gain of chromosome 7 and loss of chromosome 10 (+7/-10) [e] Methylation status as determined by qMSP and Pyrosequencing (qMSP result / pyrosequencing result).

Supplementary Table 1: Summary of in-house patient characteristics (n = 67).

Gene	Regulation in pGBM-I1 (*vs.* Normal, FDR<0.05)	Regulation in pGBM-I2 (*vs.* Normal, FDR<0.05)	Regulation in pGBM-I1 (*vs.* pGBM-I2, FDR<0.05)
VEGFA	-	-	Up
IL10	Up	-	Up
IL23A	-	-	Up
TGFB1	Up	-	Up
SPATA2	Down	Down	-
PDL1/CD274	-	-	Up
CTLA4	-	-	Up
CSF1	Up	-	Up
CCL2	Up	-	Up
CCL22	-	-	Up
CD163	Up	-	Up
MSR1	Up	-	Up

GDF15	-	-	Up
ARG1	-	Down	-
CD47	-	-	-
IL6	-	-	Up
IL6ST	Up	Up	-
JAK2	-	-	-
STAT3	Up	Up	Up
PIM1	-	-	Up
SOCS3	-	-	Up
STAT5A	-	-	Up
STAT5B	-	-	Down
CD4	Up	-	Up
ICOS	-	-	Up
FOXP3	-	-	Up

Supplementary Table 2: Regulation of immune suppressor genes in the pGBM infiltration clusters of the exploratory cohort.

Gene	Regulation in pGBM-I1 (*vs.* Normal, FDR<0.05)	Regulation in pGBM-I2 (*vs.* Normal, FDR<0.05)	Regulation in pGBM-I1 (*vs.* pGBM-I2, FDR<0.05)
IFN-γ	-	-	**Up**
IL1A	-	-	**Up**
IL2	-	-	-
IL4	-	-	-
IL7	-	-	**Up**
IL12A	-	-	**Down**
IL15	-	-	**Up**
TNF	-	-	**Up**
CD3D	-	-	**Up**
CD8A	-	-	**Up**
CD8B	-	-	**Up**
CD80	-	-	**Up**
CD86	**Up**	-	**Up**

CD40	-	-	Up
HLA-B	Up	-	Up
HLA-DRA	Up	-	Up
HLA-DBP1	Up	-	Up
B2M	Up	Up	-
RELA	Up	Up	Up
STAT1	-	Up	Down
IRAK1	Up	-	Up
STAT4	Down	Down	Up
TBX21	-	-	Up
CD226	Up	-	Up
IRF7	-	-	Up
NCR1	-	-	Up
NCR3	-	-	Up
NCR2	-	-	Up

KLRD1	-	-	**Up**
CD244	-	-	**Up**
TLR2	**Up**	-	**Up**
TLR3	**Up**	**Up**	-
TLR4	-	-	**Up**
TLR9	-	-	**Up**
TREM1	**Up**	-	**Up**
TREM2	**Up**	-	**Up**
MIF	-	-	**Up**

Supplementary Table 3: Regulation of immune effector genes in the pGBM infiltration clusters of the exploratory cohort.

				Univariable models		Multivariable model	
		Patients (n)	Events (n)	HR (95% CI)	*p*-value	HR (95% CI)	*p*-value
Molecular classif.				*p*-value of the model: 0.086			
	Classical	113	73	-		1.00 (ref)	
	Mesenchymal	137	101	-		1.71 (1.23-2.39)	**0.0016**
	Neural	73	52	-		1.21 (0.85-1.73)	0.29
	Proneural	105	75	-		1.34 (0.97-1.86)	0.080
Immune classif.				*p*-value of the model: 0.032			
	pGBM-I1	192	130	1.00 (ref)		1.00 (ref)	
	pGBM-I2	236	171	1.29 (1.02-1.61)	**0.032**	1.51(1.14-2.00)	**0.0037**

Supplementary Table 4: Univariable and multivariable Cox's proportional hazard analyses, with survival as endpoint, in the exploratory cohort.

		Patients (n)	Events (n)	Univariable models HR (95% CI)	Univariable models *p*-value	Multivariable model HR (95% CI)	Multivariable model *p*-value
Molecular classif.				*p*-value of the model: 0.033			
	Classical	32	21	1.00 (ref)		1.00 (ref)	
	Mesenchymal	35	27	1.02 (0.58-1.81)	0.94	1.30 (0.70-2.43)	0.41
	Neural	29	16	0.60 (0.31-1.15)	0.12	0.61 (0.32-1.18)	0.15
	Proneural	28	12	0.42 (0.21-0.86)	**0.018**	0.39 (0.19-0.80)	**0.010**
Immune classif.				*p*-value of the model: 0.47			
	pGBM-I1	57	35	-		1.00 (ref)	
	pGBM-I2	67	41	-		1.69 (1.00-2.84)	**0.048**

Supplementary Table 5: Univariable and multivariable Cox's proportional hazard analyses, with survival as endpoint, in the validation cohort.

		Patients (n)	Events (n)	Univariable models HR (95% CI)	Univariable models *p*-value	Multivariable model HR (95% CI)	Multivariable model *p*-value
Molecular classif.				*p*-value of the model: 0.17			
	Classical	145	94	-		1.00 (ref)	
	Mesenchymal	172	128	-		1.63 (1.21-2.18)	**0.0012**
	Neural	102	68	-		1.09 (0.80-1.49)	0.59
	Proneural	133	87	-		1.09 (0.81-1.47)	0.57
Immune classif.				*p*-value of the model: 0.025			
	pGBM-I1	249	165	1.00 (ref)		1.00 (ref)	
	pGBM-I2	303	212	1.26 (1.03-1.55)	**0.025**	1.55 (1.21-1.97)	**4.8e-4**
Cohort				*p*-value of the model: 0.0022			
	In-house	36	30	1.00 (ref)		1.00 (ref)	
	TCGA	392	271	0.075 (0.49-1.04)		0.76 (0.51-1.11)	0.15
	Rembrandt	124	76	0.0013 (0.33-0.76)		0.53 (0.34-0.82)	**0.0043**

Supplementary Table 6: Univariable and multivariable Cox's proportional hazard analyses, with survival as endpoint, in the pooled exploratory and validation cohorts.

	pGBM-I1				pGBM-I2			
	Patients (n)	Events (n)	HR (95% CI)	*p*-value	Patients (n)	Events (n)	HR (95% CI)	*p*-value
Molecular classif.								
Classical	51	30	1.00 (ref)		94	64	1.00 (ref)	
Mesenchymal	140	100	1.55 (1.03-2.33)	**0.037**	32	28	1.81 (1.15-2.86)	**0.011**
Neural	34	18	0.87 (0.48-1.57)	0.64	68	50	1.19 (0.82-1.73)	0.36
Proneural	24	17	1.52 (0.84-2.78)	0.17	109	70	1.02 (0.73-1.44)	0.90
Cohort								
In-house	19	15	1.00 (ref)		17	15	1.00 (ref)	
TCGA	173	115	0.70 (0.41-1.19)	0.19	219	156	0.84 (0.48-1.47)	0.55
Rembrandt	57	35	0.52 (0.28-0.96)	**0.037**	67	41	0.56 (0.30-1.04)	0.066

Supplementary Table 7: Effect of molecular subtypes on survival in pGBM-I1 and pGBM-I2, in the pooled exploratory and validation cohorts.

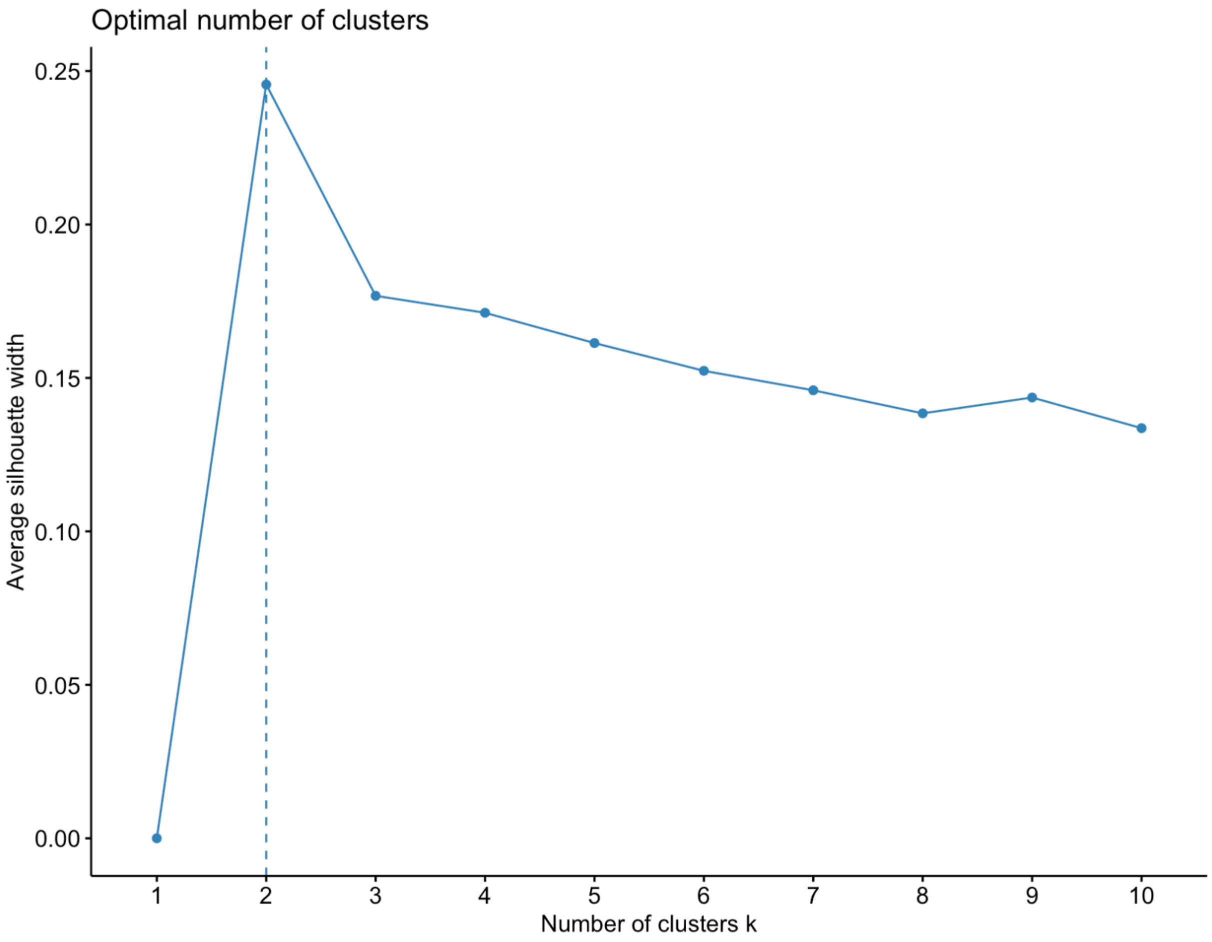

Supplementary Figure 1: Selection of the number of pGBM infiltration clusters in the exploratory cohort. The silhouette plot identifies the optimal number of clusters, which maximizes the average silhouette width over a range of possible values (k = 1 to 10).

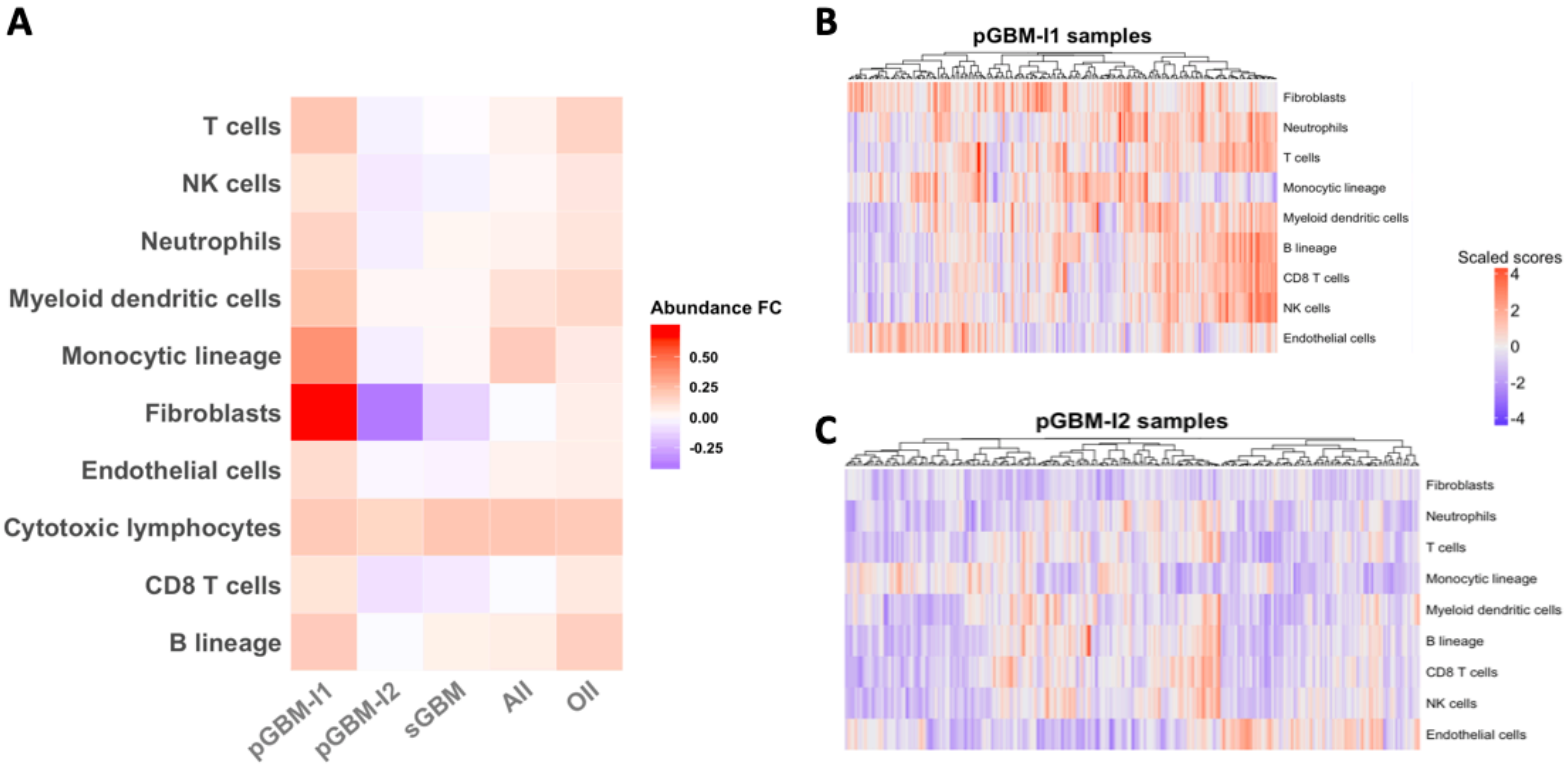

Supplementary Figure 2: Estimation of tumor infiltration across samples of the exploratory cohort. (A) Abundance fold-changes (FC), *i.e.* average infiltration scores in tumors - averages infiltration score in normal samples. Red color indicates a higher infiltration in tumors, compared to normal, while blue color underlines a lower infiltration in tumors. Heatmaps of MCPcounter scores in (B) pGBM-I1 and (C) pGBM-I2 samples. Columns are ordered using a hierarchical clustering procedure, using 1-Pearson's correlation as a measure of distance between samples.

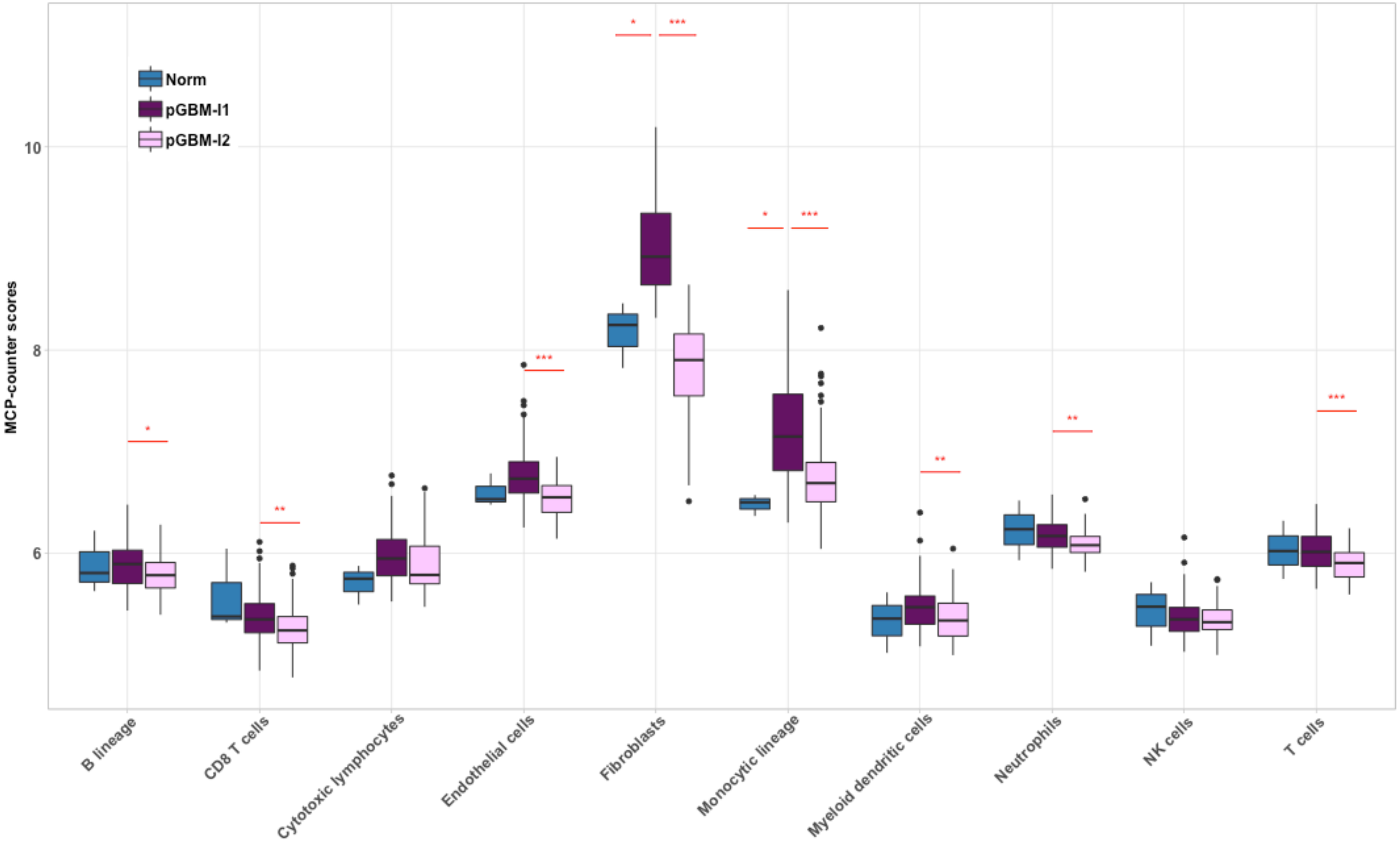

Supplementary Figure 3: Differential abundance of immune and stromal cell populations between pGBM-I1 (n=57) and pGBM-I2 (n=67), in the validation dataset. FDR values from limma (Benjamini-Hochberg procedure) are indicated for each comparison. Legend: (*) FDR<0.05, (**) FDR<0.01, (***) FDR<0.001.

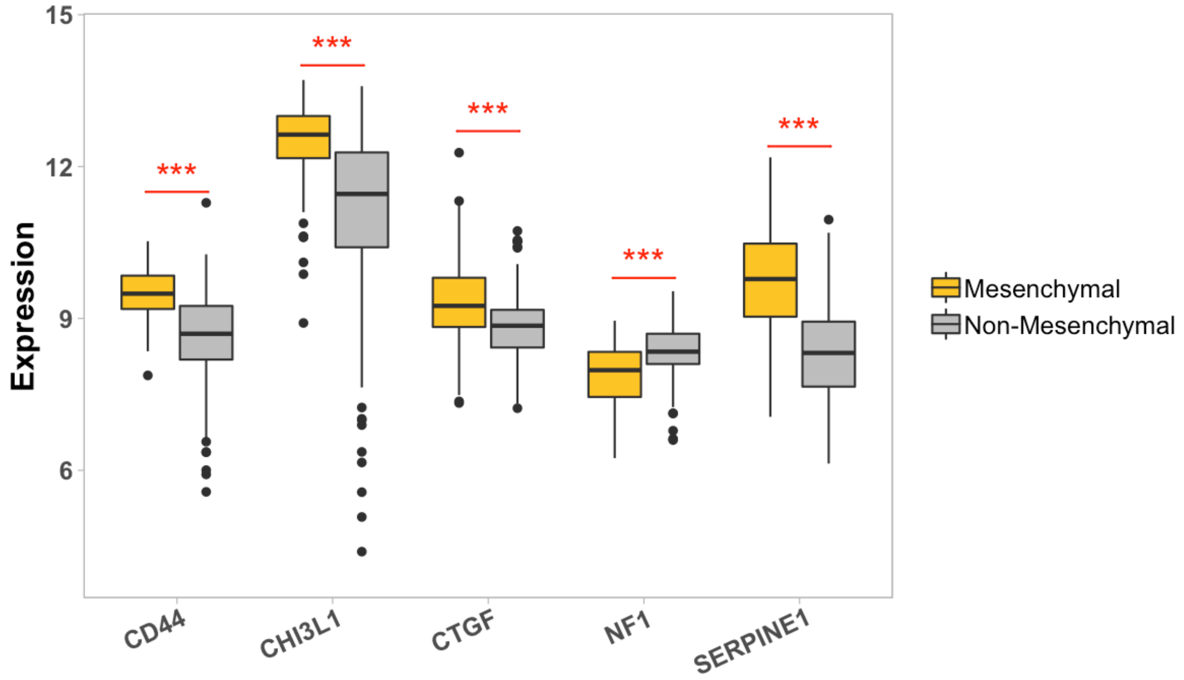

Supplementary Figure 4: Expression levels of selected genes in the mesenchymal subtype *vs.* other subtypes (neural, proneural and classical) in the exploratory cohort. FDR values from limma (Benjamini-Hochberg procedure) are indicated for each comparison. Legend: (*) FDR<0.05, (**) FDR<0.01, (***) FDR<0.001.

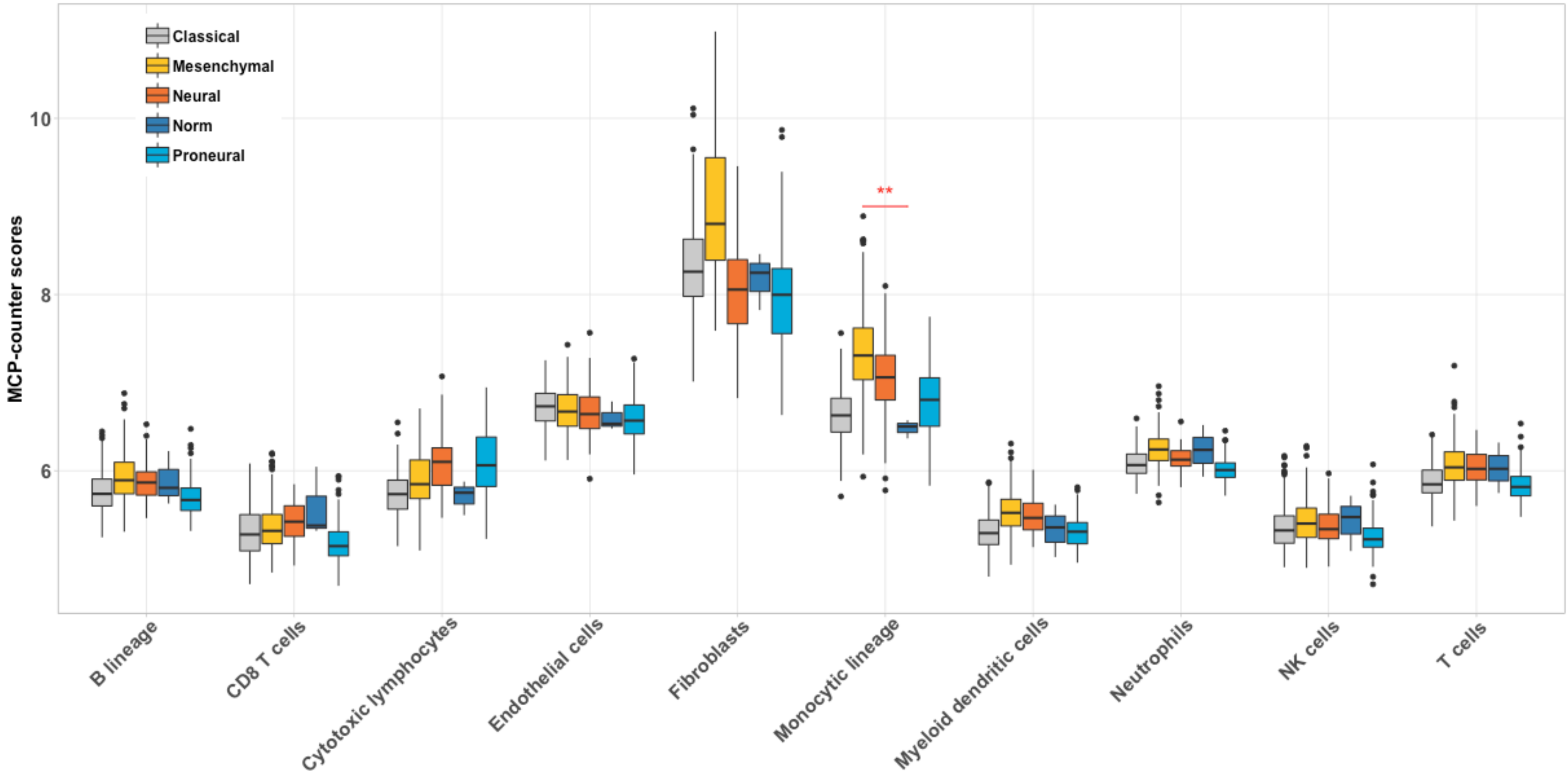

Supplementary Figure 5: Comparison of abundance of immune and stromal cell populations in the pGBM molecular subtypes (exploratory cohort).

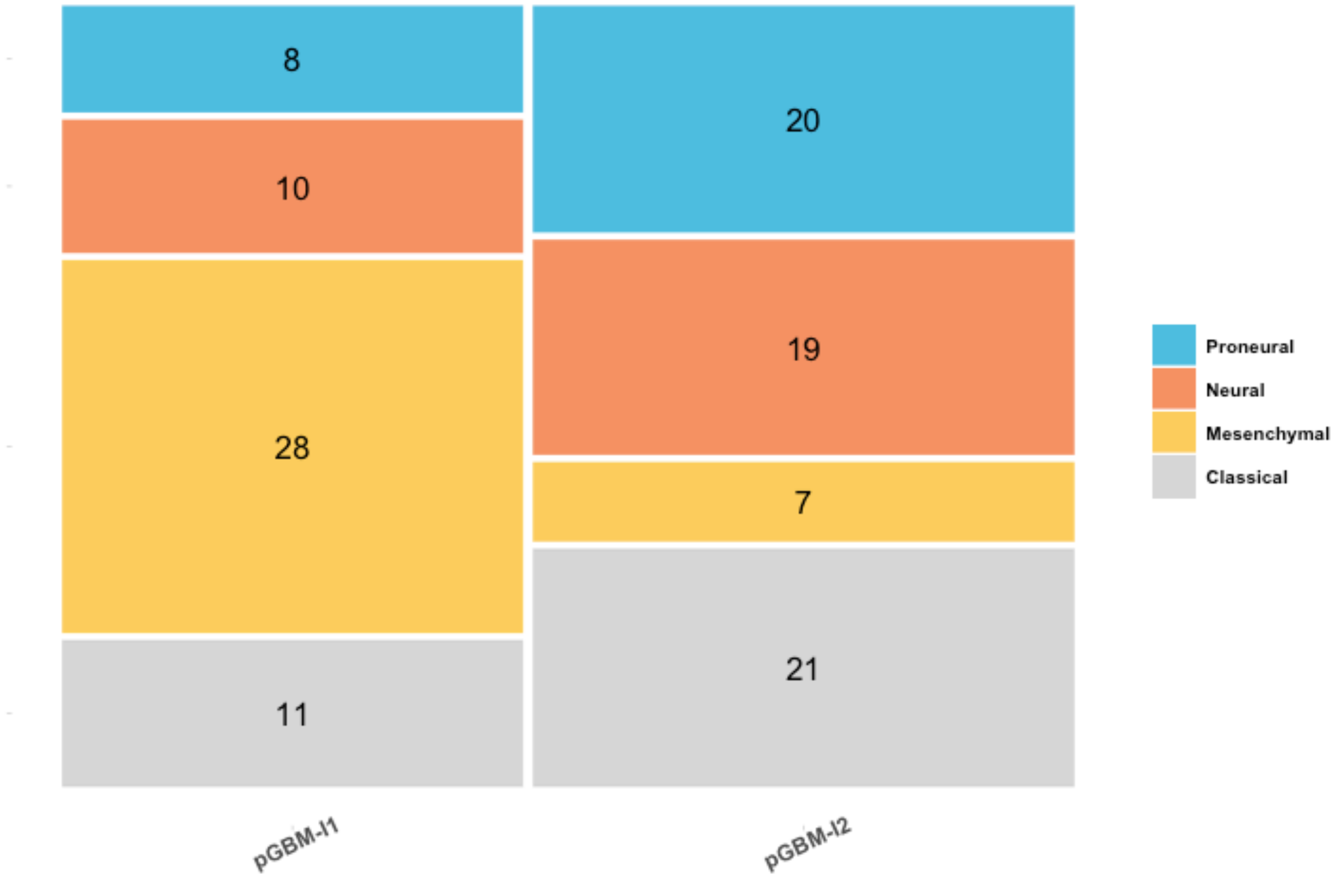

Supplementary Figure 6: Molecular characterization of the pGBM-I1 (n=57) and pGBM-I2 (n=67) clusters according to the Verhaak's signatures in the validation dataset.

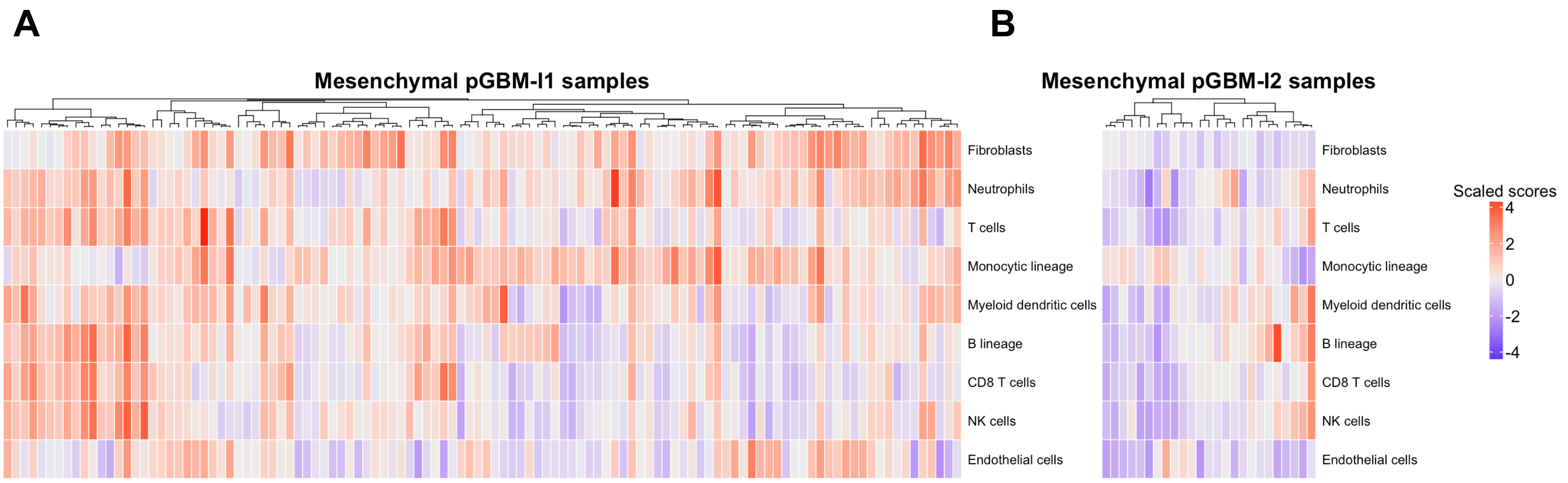

Supplementary Figure 7: Heatmaps of MCPcounter scores in mesenchymal tumors of the exploratory cohort (A) pGBM-I1 and (B) pGBM-I2. Columns are ordered using a hierarchical clustering procedure, using 1-Pearson's correlation as a measure of distance between samples.

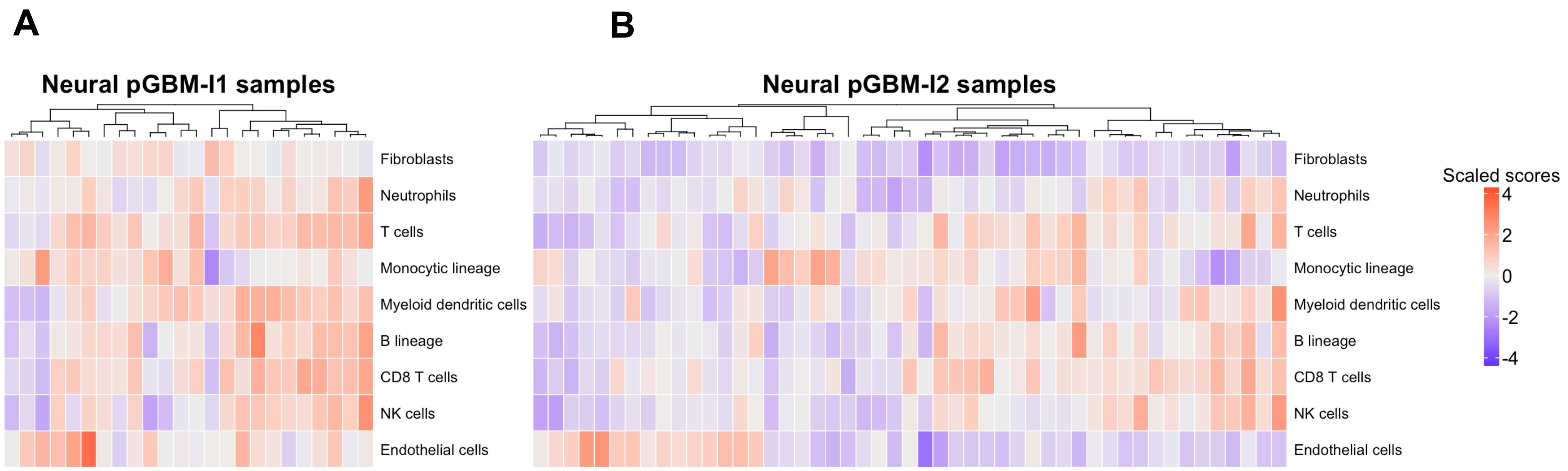

Supplementary Figure 8: Heatmaps of MCPcounter scores in neural tumors of the exploratory cohort (A) pGBM-I1 and (B) pGBM-I2. Columns are ordered using a hierarchical clustering procedure, using 1-Pearson's correlation as a measure of distance between samples.

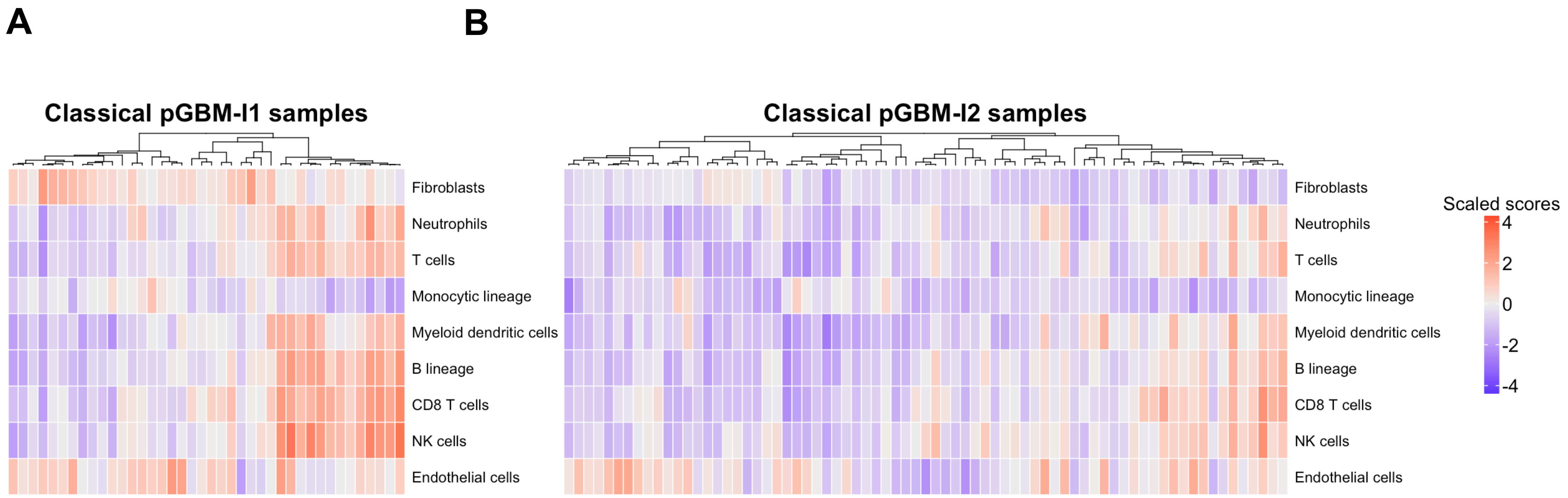

Supplementary Figure 9: Heatmaps of MCPcounter scores in classical tumors of the exploratory cohort (A) pGBM-I1 and (B) pGBM-I2. Columns are ordered using a hierarchical clustering procedure, using 1-Pearson's correlation as a measure of distance between samples.

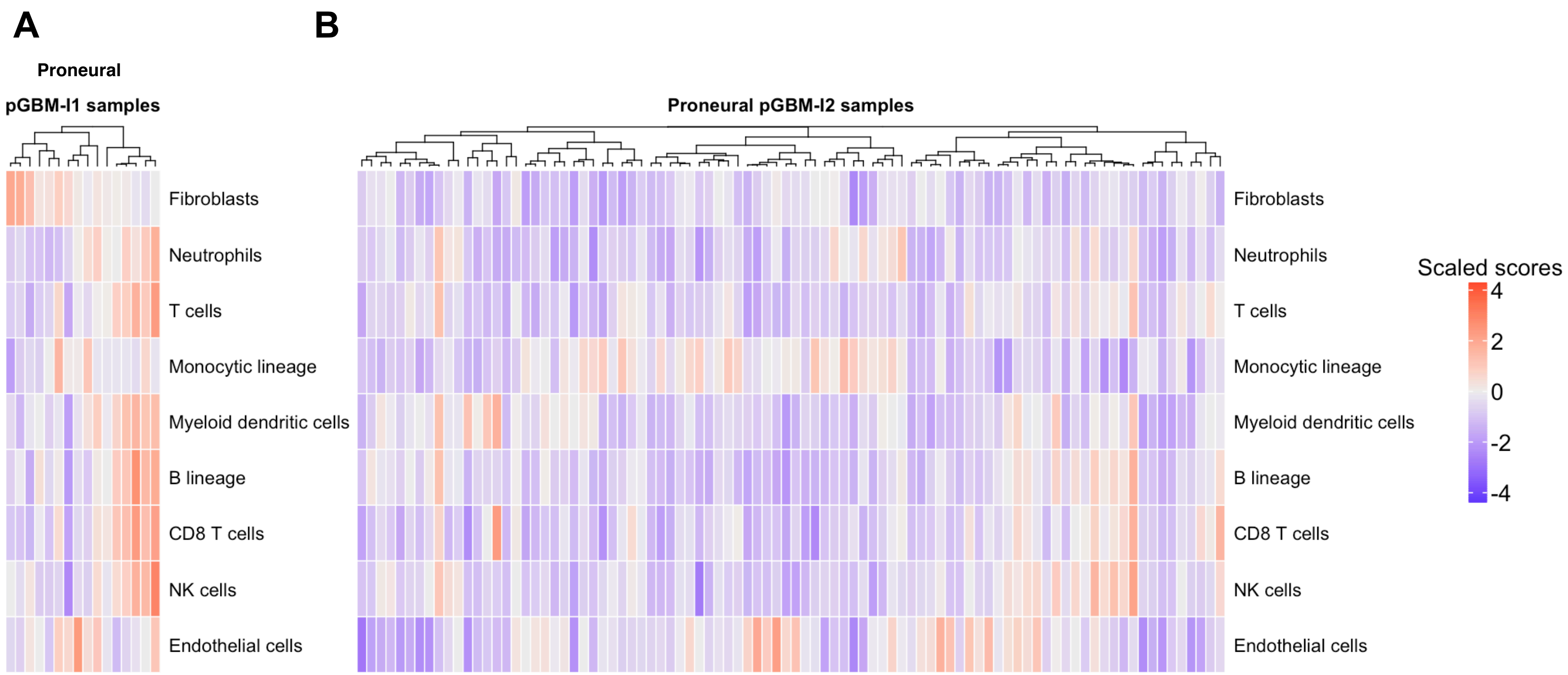

Supplementary Figure 10: Heatmaps of MCPcounter scores in proneural tumors of the exploratory cohort (A) pGBM-I1 and (B) pGBM-I2. Columns are ordered using a hierarchical clustering procedure, using 1-Pearson's correlation as a measure of distance between samples.

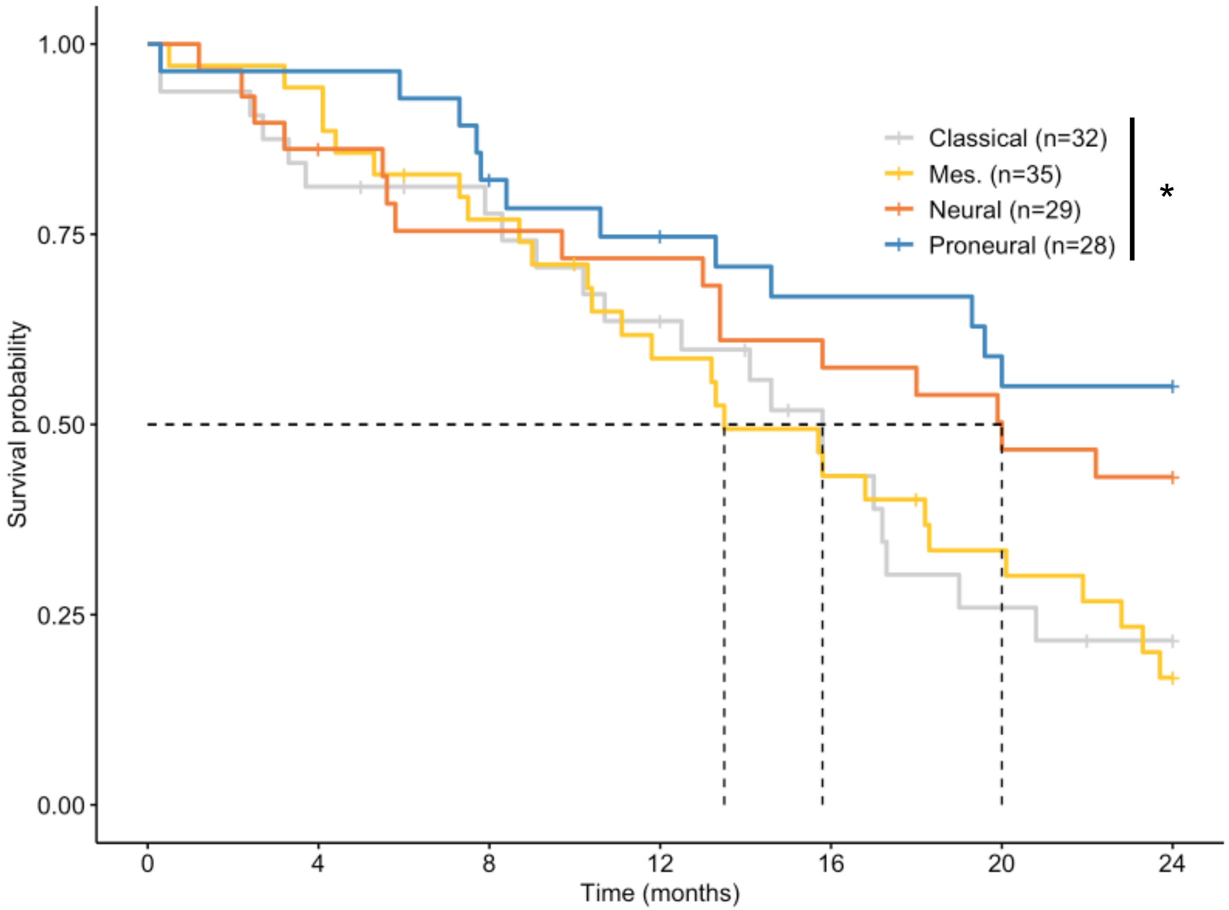

Supplementary Figure 11: Kaplan-Meier curves modeling the effect of GBM molecular classification on survival in the validation cohort.

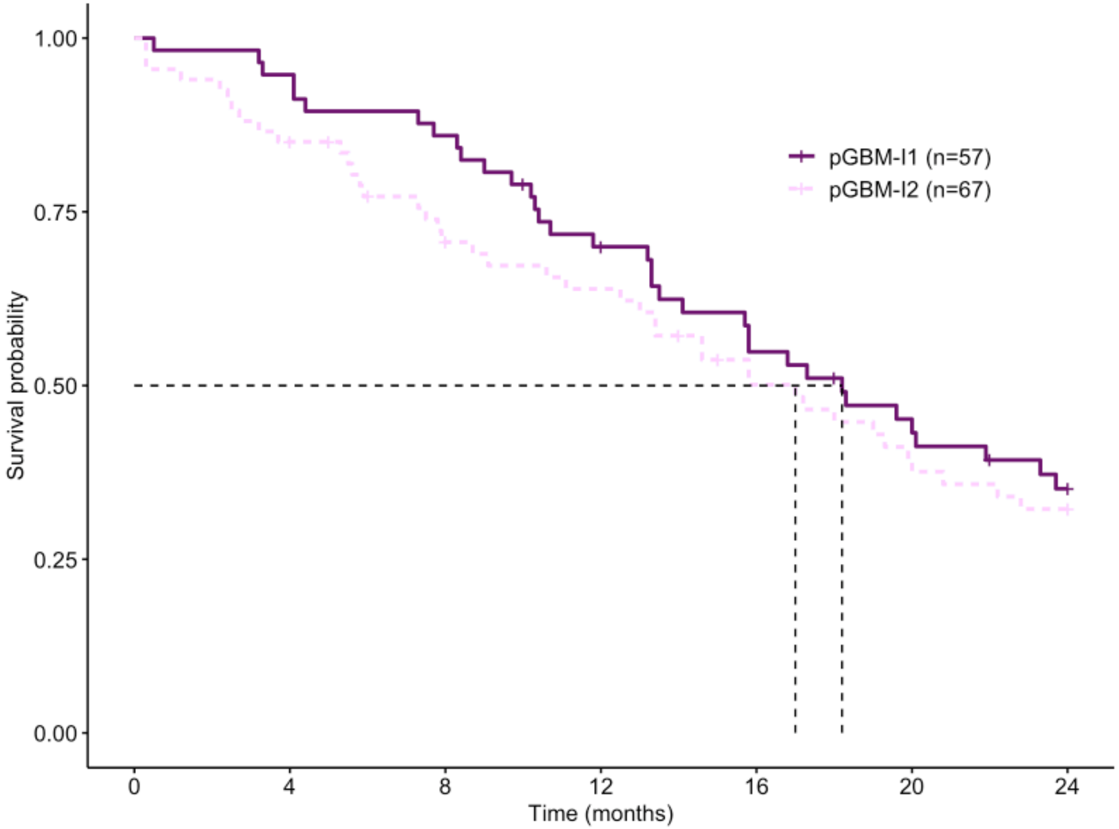

Supplementary Figure 12: Kaplan-Meier curves modeling the effect of tumor infiltration on overall survival in the validation cohort.

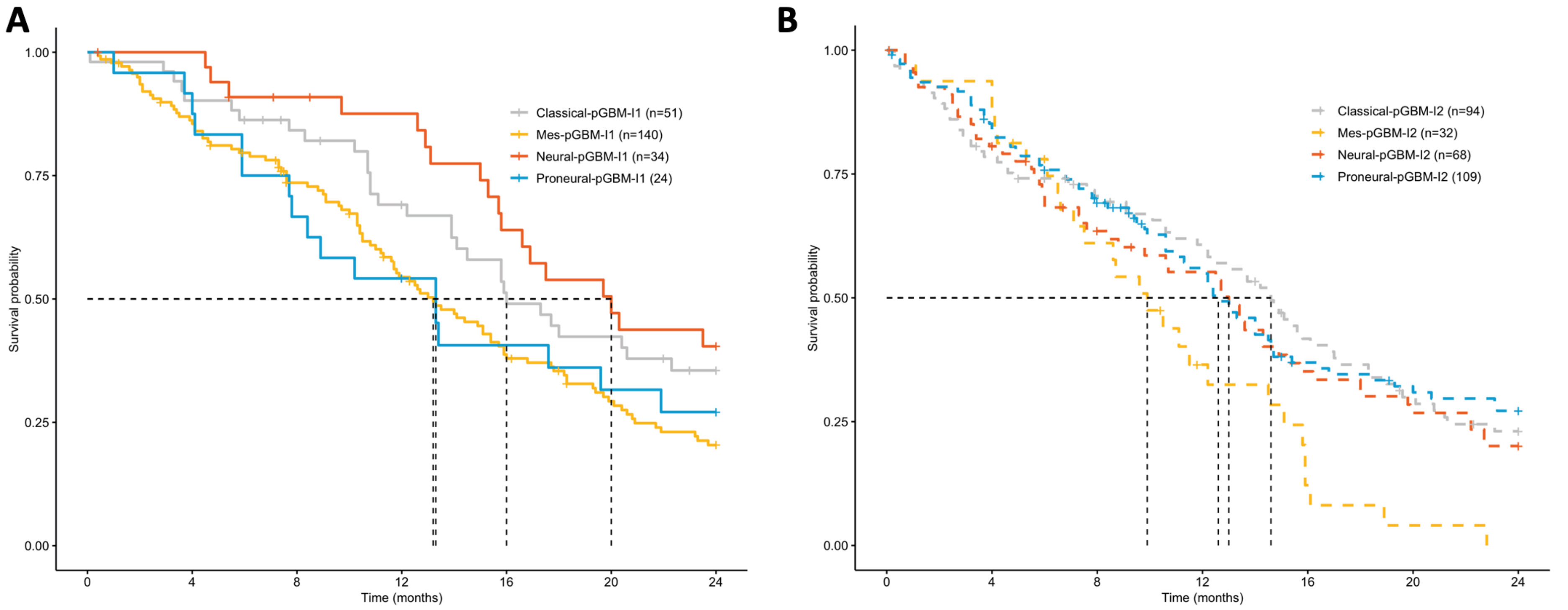

Supplementary Figure 13: Effect of molecular subtypes on survival, in the pooled exploratory and validation cohorts, in (A) pGBM-I1 and (B) pGBM-I2.
